# The Performance of AI in Dermatology Exams: The Exam Success and Limits of ChatGPT

**DOI:** 10.1111/jocd.70244

**Published:** 2025-05-19

**Authors:** Neşe Göçer Gürok, Savaş Öztürk

**Affiliations:** ^1^ Department of Dermatology Elazığ Fethi Sekin City Health Application and Research Center, University of Health Sciences Elazig Turkey

**Keywords:** AI, artificial intelligence, ChatGPT, dermatology education, exam

## Abstract

**Background:**

Artificial intelligence holds significant potential in dermatology.

**Objectives:**

This study aimed to explore the potential and limitations of artificial intelligence applications in dermatology education by evaluating ChatGPT's performance on questions from the dermatology residency exam.

**Method:**

In this study, the dermatology residency exam results for ChatGPT versions 3.5 and 4.0 were compared with those of resident doctors across various seniority levels. Dermatology resident doctors were categorized into four seniority levels based on their education, and a total of 100 questions—25 multiple‐choice questions for each seniority level—were included in the exam. The same questions were also administered to ChatGPT versions 3.5 and 4.0, and the scores were analyzed statistically.

**Results:**

ChatGPT 3.5 performed poorly, especially when compared to senior residents. Second (*p* = 0.038), third (*p* = 0.041), and fourth‐year senior resident physicians (*p* = 0.020) scored significantly higher than ChatGPT 3.5. ChatGPT 4.0 showed similar performance compared to first‐ and third‐year senior resident physicians, but performed worse in comparison to second (*p* = 0.037) and fourth‐year senior resident physicians (*p* = 0.029). Both versions scored lower as seniority and exam difficulty increased. ChatGPT 3.5 passed the first and second‐year exams but failed the third and fourth‐year exams. ChatGPT 4.0 passed the first, second, and third‐year exams but failed the fourth‐year exam. These findings suggest that ChatGPT was not on par with senior resident physicians, particularly on topics requiring advanced knowledge; however, version 4.0 proved to be more effective than version 3.5.

**Conclusion:**

In the future, as ChatGPT's language support and knowledge of medicine improve, it can be used more effectively in educational processes.

## 1 Introduction

Artificial intelligence, particularly large language models (LLMs), is increasingly influencing the medical field. Rapid advancements in AI models are creating new opportunities in medical education by responding to user inquiries, organizing medical information, and providing clinical decision support [1]. Notably, advanced language models like the Chat Generative Pretrained Transformer (ChatGPT), developed by OpenAI (San Francisco, CA, USA), version 3.5 first released in 2022 and version 4.0 in 2023, possess significant potential for personalized access to information and educational resources for medical students and healthcare professionals [2]. As a result, current studies are underway in the healthcare industry to evaluate the exam performance and clinical approach of ChatGPT [3, 4].

ChatGPT also shows promise in dermatology applications such as patient education, clinical decision support, and teledermatology. Dermatology training requires extensive knowledge and quick decision‐making skills. While artificial intelligence is gaining popularity, evidence supporting its use in dermatology remains limited [5]. Notably, no studies have been conducted on ChatGPT in the field of dermatology in Turkish. As this will be the first study to address this gap, its results could be significant. In this context, assessing ChatGPT's performance on dermatology resident physician exam questions will be valuable for understanding the potential of artificial intelligence applications in medical education, the effectiveness of integrating such systems into healthcare services, and their limitations. For this purpose, we posed the dermatology exam questions to ChatGPT versions 3.5 and 4.0 to comprehensively analyze the performance of ChatGPT in diagnostic reasoning, clinical decision making, and treatment, compare the answers of ChatGPT with those of dermatology residents at different years of seniority, and analyze the potential of ChatGPT in medical education.

## 2 Methods

**FIGURE 1:**
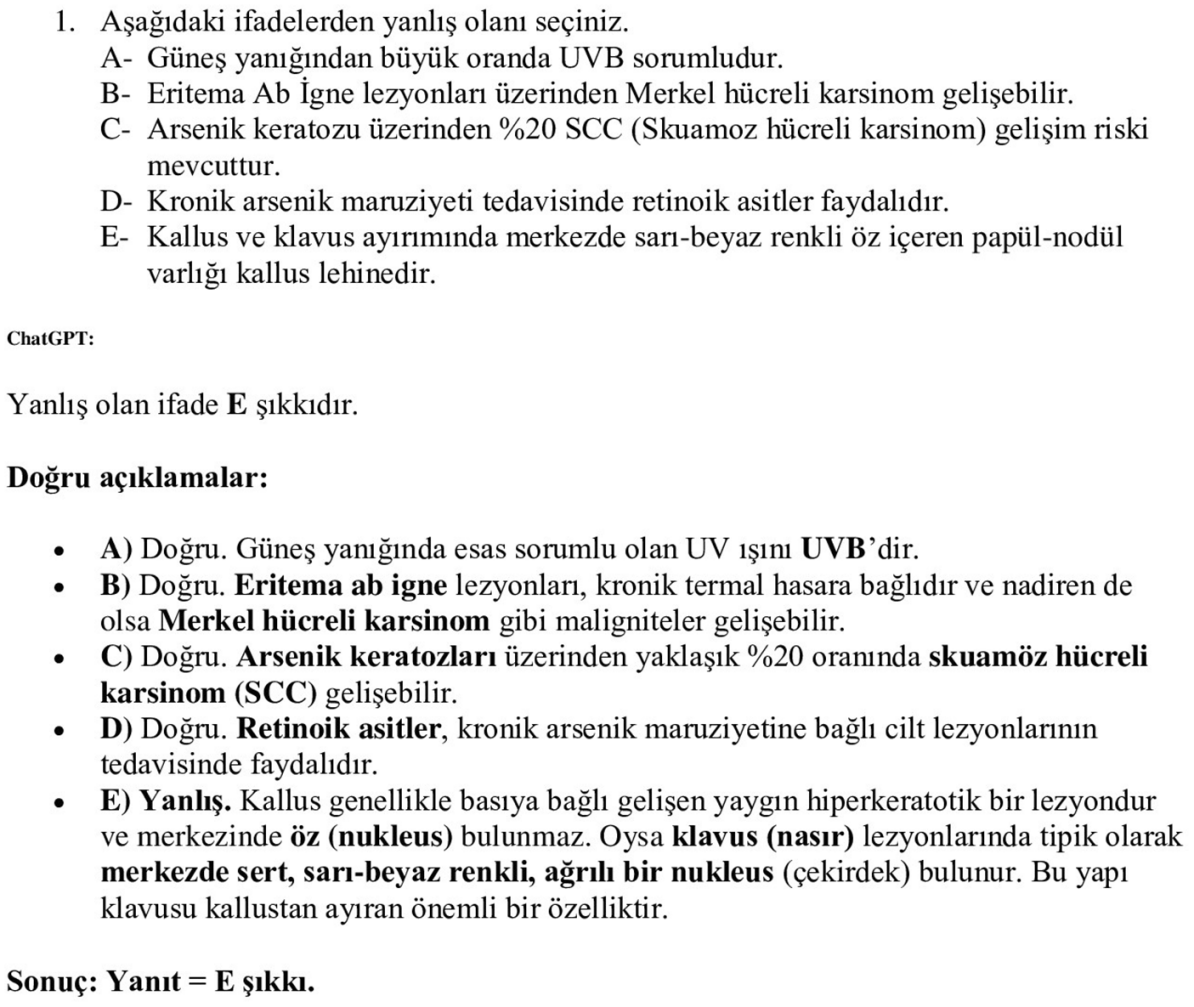
Example of a prompt we used for getting response from ChatGPT4.0.

In the study, 100 questions were created in Turkish, divided into four groups of 25 questions each, based on the seniority of dermatology resident physicians. The questions, developed by dermatologist faculty members, did not include photographs and required basic knowledge, clinical practice, and complex thinking skills. The Health Sciences University Measurement and Evaluation Board Examination Committee approved the questions. Resident physicians were categorized according to their seniority: 0–12 months of residency was considered the first year, 12–24 months the second year, 24–36 months the third year, and over 36 months the fourth year. Twelve first‐year, five second‐year, five third‐year, and three fourth‐year resident physicians from the Health Sciences University Fethi Sekin Urban Hospital Dermatology Clinic participated in the study. Each group was tasked with answering 25 multiple‐choice questions with five options based on their seniority, and they were instructed to respond individually, simultaneously, under the same conditions, and under supervision. A prospective study was conducted in January 2025 using common LLMs, OpenAI's ChatGPT version 3.5 (GPT‐3.5) and ChatGPT version 4.0 (GPT‐4.0). The same questions were posed to these models in Turkish, and their responses were evaluated against the answer key (Figure 1). Questions are prompted as in articles similar to ChatGPT [6]. The mean scores of the resident physicians were compared with those of ChatGPT. The SBU Fethi Sekin City Hospital Non‐Interventional Research Ethics Committee approved the study, which was conducted in accordance with Good Clinical Practice guidelines and the principles of the Helsinki Declaration.

### 2.1 Statistical Analysis

The study data were analyzed using SPSS version 25.0. Descriptive statistics are presented as Mean ± SD. Normality of the data was assessed with the Shapiro–Wilk test. The Mann–Whitney *U* test, a nonparametric method, was used to determine whether significant differences existed between the groups. A *p* < 0.05 was considered statistically significant.

## 3 Results

In the study, the scores of ChatGPT versions 3.5 and 4.0 on various levels of Turkish dermatology residency exams were compared to those of resident physicians (Table 1). ChatGPT 3.5's performance was lower, particularly when compared to senior residents. For instance, the scores of second‐year (*p* = 0.038), third‐year (*p* = 0.041), and fourth‐year (*p* = 0.020) resident physicians were significantly higher than those of ChatGPT 3.5. Meanwhile, ChatGPT 4.0's performance was comparable to that of third‐year resident physicians (*p* = 0.073) but lower than that of second‐year (*p* = 0.037) and fourth‐year resident physicians (*p* = 0.029). Furthermore, the scores of ChatGPT 3.5 were lower than those of first‐year resident physicians, while ChatGPT 4.0 scored higher; however, these differences were not statistically significant (*p* = 0.167, 0.358). Both GPT 3.5 and 4.0 experienced a decline in scores as the year and exam difficulty increased. In the exams, a passing grade is 60/100; therefore, GPT 3.5 passed the first and second years but failed the third and fourth‐year exams. GPT‐4.0 passed the first, second, and third‐year exams but failed the fourth‐year exam. These findings demonstrate that ChatGPT was not on par with senior resident physicians in Turkish, especially in subjects requiring advanced knowledge, while the performance of version 4.0 was superior compared to that of version 3.5.

**TABLE 1:** Inter‐group comparison.

Variable	Score (mean ± SD)	*p*
1st year dermatology resident	70.33 ± 5.51	
vs ChatGPT 3.5	68.00 ± 0.00	0.167
vs ChatGPT 4.0	72.00 ± 0.00	0.358
2nd year dermatology resident	74.40 ± 2.19	
vs ChatGPT 3.5	64.00 ± 0.00	0.038*
vs ChatGPT 4.0	68.00 ± 0.00	0.037*
3rd year dermatology resident	65.00 ± 1.96	
vs ChatGPT 3.5	52.00 ± 0.00	0.041*
vs ChatGPT 4.0	64.00 ± 0.00	0.073
4th year dermatology resident	64.00 ± 4.00	
vs ChatGPT 3.5	40.00 ± 0.00	0.020*
vs ChatGPT 4.0	48.00 ± 0.00	0.029*

* *p* < 0.05 indicates statistically significant difference.

## 4 Discussion

Rapid advances in artificial intelligence and natural language processing (NLP) have opened up new applications in medical sciences. The capability of these LLMs to answer questions and make accurate inferences based on clinical knowledge has become increasingly significant [7].

In particular, advanced language models like ChatGPT are crucial due to their potential in dermatology. As an artificial intelligence model trained on extensive datasets, ChatGPT can assist in answering dermatological questions and counseling patients [8]. Therefore, we found that ChatGPT‐4.0 outperformed ChatGPT‐3.5, that the success rate of both versions decreased as the difficulty of the questions increased with seniority, and that while they showed similar performance with resident physicians at certain seniority levels, both versions did worse than resident physicians at others.

Several studies have assessed the performance of ChatGPT in dermatology. In one study, three Dermatology Specialist Certification Exams, each consisting of 120 multiple‐choice questions in English and Polish, were used to evaluate the performances of ChatGPT‐3.5 and ChatGPT‐4. ChatGPT‐4 surpassed the 60% passing rate in each test, achieving at least 80% and 70% correct answers in the English and Polish versions, respectively. Reports indicated that ChatGPT's dermatology knowledge was high, with ChatGPT‐4 demonstrating significantly better performance than ChatGPT‐3.5 [9].

Another study assessing ChatGPT's performance in dermatology residency exams focused on the AI model's ability to answer clinical questions. In a test consisting of 84 multiple‐choice questions, ChatGPT version 3.5 achieved a success rate of 63%, while ChatGPT version 4 reached 90%, exceeding the passing grade of 70%–72% for the dermatology residency exam [10].

However, some papers have reported that using artificial intelligence in dermatology poses ethical and practical limitations, and that additional research is required on the accuracy and reliability of ChatGPT in dermatological diagnosis and treatment [11].

A study compared the responses of ChatGPT versions 3.5 and 4.0 to multiple‐choice and case‐based questions with those of pediatric dermatologists. It found that pediatric dermatology clinicians generally outperformed both versions of ChatGPT; however, the performance of ChatGPT‐4.0 was comparable in some areas. The study emphasized that AI tools can assist clinicians with medical knowledge and decision‐making, but clinician supervision is essential [12].

One reason ChatGPT's performance was lower in our study compared to previously mentioned studies could be that the exam questions were considered quite difficult relative to past resident physician exams. Additionally, since dermatology is a vast field with a significant theoretical load, the questions may have focused on specific areas where ChatGPT's data was incomplete. Upon reviewing the answers, we found that the questions both versions answered incorrectly were similar. Furthermore, because ChatGPT's command of the Turkish language is not as strong as its command of English, it struggled with complex questions. A multilingual learning study analyzed ChatGPT's performance on seven tasks across 37 languages and concluded that its performance in languages other than English is generally weaker, highlighting a need for further development in multilingual learning [13]. Similarly, a recent study reported that the Hindu translation by ChatGPT was unsuitable for communication with patients [14]. In our study, ChatGPT's scores decreased as question difficulty increased with higher seniority. This illustrates that while ChatGPT can facilitate learning and decision‐making at basic levels, its advantages diminish when high‐level reasoning and contextual judgment are necessary. Although the scores of resident physicians also declined with increased question difficulty, the extreme scores observed at certain seniority levels may be attributed to variations in learning and thinking skills as well as differences in individual achievement levels.

## 5 Conclusion

The results of this study indicate that ChatGPT can be utilized in Turkish dermatology exams; however, its performance varies based on the knowledge level of resident physicians. Specifically, the knowledge and experience of senior residents surpass the model's accuracy. ChatGPT performed better in basic dermatology, general dermatological diagnosis, and clinical properties of diseases but showed limited success in more advanced clinical applications, such as complex clinical decision‐making, individual treatment planning based on patient traits, and rare or atypical dermatological conditions.

Although ChatGPT version 4.0 showed greater success than version 3.5, it was concluded that it cannot fully replace human expertise in critical stages of medical education at this time. In the future, when Turkish language support and ChatGPT's medical knowledge are further enhanced, it can be used more effectively in educational processes.

### 5.1 Limitations

The small sample size (25 residents) limits the generalizability of the findings. Additionally, using only multiple‐choice questions without photographs may have led to an insufficient assessment in a field that requires visual information, such as dermatology. The study focused exclusively on ChatGPT versions 3.5 and 4.0, without comparing the results to those of other significant language models. The language model was evaluated only in Turkish and cannot be generalized to other languages. Furthermore, the study was a short‐term prospective investigation; therefore, it cannot offer insights into the long‐term performance and development of the models. More comprehensive studies should be conducted to assess the future performance and clinical benefits of these continually evolving models.

## Author Contributions

N.G.G. conceptualized, planned and executed the study, conducted the clinical experiments, researched the data, performed statistical analyses and wrote the manuscript. S.Ö. planned and executed the study, researched the data and wrote parts of the manuscript.

## Ethics Statement

Non‐Interventional Research Ethics Committee approved the study.

## Conflicts of Interest

The authors declare no conflicts of interest.

## Data Availability

The data that support the findings of this study are available on request from the corresponding author. The data are not publicly available due to privacy or ethical restrictions.

## References

[1] Y. Ahmed , “Utilization of ChatGPT in Medical Education: Applications and Implications for Curriculum Enhancement,” Acta Informatica Medica 31, no. 4 (2023): 300–305. 38379690 10.5455/aim.2023.31.300-305 PMC10875960

[2] A. E. Günay , A. Özer , A. Yazıcı , and G. Sayer , “Comparison of ChatGPT Versions in Informing Patients With Rotator Cuff Injuries,” JSES International 8, no. 5 (2024): 1016–1018. 39280147 10.1016/j.jseint.2024.04.016 PMC11401580

[3] A. Meyer , J. Riese , and T. Streichert , “Comparison of the Performance of GPT‐3.5 and GPT‐4 With That of Medical Students on the Written German Medical Licensing Examination: Observational Study,” JMIR Medical Education 10 (2024): e50965. 38329802 10.2196/50965 PMC10884900

[4] P. A. Massey , C. Montgomery , and A. S. Zhang , “Comparison of ChatGPT‐3.5, ChatGPT‐4, and Orthopaedic Resident Performance on Orthopaedic Assessment Examinations,” Journal of the American Academy of Orthopaedic Surgeons 23, no. 31 (2023): 1173–1179. 10.5435/JAAOS-D-23-00396 PMC10627532 37671415

[5] P. Goktas and A. Grzybowski , “Assessing the Impact of ChatGPT in Dermatology: A Comprehensive Rapid Review,” Journal of Clinical Medicine 19, no. 13 (2024): 5909. 10.3390/jcm13195909 PMC11477344 39407969

[6] P. K. Sarangi and H. Mondal , “Response Generated by Large Language Models Depends on the Structure of the Prompt,” Indian Journal of Radiology & Imaging 34, no. 3 (2024): 574–575, 10.1055/s-0044-1782165. 38912242 PMC11188729

[7] A. Gilson , C. W. Safranek , T. Huang , et al., “How Does ChatGPT Perform on the United States Medical Licensing Examination (USMLE)? The Implications of Large Language Models for Medical Education and Knowledge Assessment,” JMIR Medical Education 9 (2023): e45312. 36753318 10.2196/45312 PMC9947764

[8] N. Kluger , “Potential Applications of ChatGPT in Dermatology,” Journal of the European Academy of Dermatology and Venereology 37 (2023): e941–e942. 37102458 10.1111/jdv.19152

[9] M. Lewandowski , P. Łukowicz , D. Świetlik , and W. Barańska‐Rybak , “ChatGPT‐3.5 and ChatGPT‐4 Dermatological Knowledge Level Based on the Specialty Certificate Examination in Dermatology,” Clinical and Experimental Dermatology 49, no. 7 (2024): 686–691. 37540015 10.1093/ced/llad255

[10] L. Passby , N. Jenko , and A. Wernham , “Performance of ChatGPT on Specialty Certificate Examination in Dermatology Multiple‐Choice Questions,” Clinical and Experimental Dermatology 49, no. 7 (2024): 722–727. 37264670 10.1093/ced/llad197

[11] Z. Zhang , J. Zhang , L. Duan , and C. Tan , “ChatGPT in Dermatology: Exploring the Limited Utility Amidst the Tech Hype,” Frontiers in Medicine 10 (2024): 1308229. 38283041 10.3389/fmed.2023.1308229 PMC10811787

[12] C. Y. Huang , E. Zhang , M. C. Caussade , T. Brown , G. Stockton Hogrogian , and A. C. Yan , “Pediatric Dermatologists Versus AI Bots: Evaluating the Medical Knowledge and Diagnostic Capabilities of ChatGPT,” Pediatric Dermatology 5, no. 41 (2024): 831–834. 10.1111/pde.15649 38721744

[13] V. D. Lai , N. T. Ngo , A. P. B. Veyseh , et al., “ChatGPT Beyond English: Towards a Comprehensive Evaluation of Large Language Models in Multilingual Learning,” arXiv 12 (2023): 5613.

[14] P. K. Sarangi , A. Lumbani , M. S. Swarup , et al., “Assessing Chatgpt's Proficiency in Simplifying Radiological Reports for Healthcare Professionals and Patients,” Cureus 12, no. 15 (2023): e50881. 10.7759/cureus.50881 PMC10799309 38249202

